# Fabrication of a Cancer Cell Aggregate Culture Device That Facilitates Observations of Nutrient and Oxygen Gradients

**DOI:** 10.3390/mi15060689

**Published:** 2024-05-24

**Authors:** Maho Kaminaga, Shuta Otomo, Seisyu Tsunozaki, Tetuya Kadonosono, Toru Omata

**Affiliations:** 1 Department of Mechanical Engineering, National Institute of Technology, Toyota Campus, 2-1 Eisei-cho, Toyota 471-0067, Aichi, Japan; 2 Department of Mechanical Engineering, Tokyo Institute of Technology, 4259 Nagatsuta-cho, Midori ku, Yokohama 226-0026, Kanagawa, Japanomata.t.aa@m.titech.ac.jp (T.O.); 3 Department of Life Science & Technology, Tokyo Institute of Technology, 4259 Nagatsuta-cho, Midori ku, Yokohama 226-0026, Kanagawa, Japan; tetsuyak@bio.titech.ac.jp

**Keywords:** cell spheroid, three-dimensional culture, cancer cell, oxygen gradient, nutrient gradient

## Abstract

Three-dimensional cell culture spheroids are commonly used for drug evaluation studies because they can produce large quantities of homogeneous cell aggregates. As the spheroids grow, nutrients supplied from outer spheroid regions render the inner spheroid areas hypoxic and hyponutrient, which makes them unobservable through confocal microscopy. In this study, we fabricated a cancer cell aggregate culture device that facilitates the observation of nutrient and oxygen gradients. An alginate gel fiber was created in the cell culture chamber to ensure a flow path for supplying the culture medium. A gradient of nutrients and oxygen was generated by positioning the flow channel close to the edge of the chamber. We devised a fabrication method that uses calcium carbonate as a source of Ca^2+^ for the gelation of sodium alginate, which has a slow reaction rate. We then cultured a spheroid of HCT116 cells, which were derived from human colorectal carcinoma using a fluorescent ubiquitination-based cell cycle indicator. Fluorescence observation suggested the formation of a hypoxic and hyponutrient region within an area approximately 500 µm away from the alginate gel fiber. This indicates the development of a cancer cell aggregate culture device that enables the observation of different nutrition and oxygen states.

## 1. Introduction

Three-dimensional (3D) cell culture spheroids have become a standard cell model for drug evaluation studies because they can produce large quantities of homogeneous cell aggregates. Several methods exist for spheroid formation. One of these methods uses a hemispherical culture chamber whose inner surface has been treated to reduce cell adhesion; cells that settle at the bottom of the chamber use each other as scaffolds to aggregate into spheroids [1]. Because nutrients are supplied from regions outside the spheroids, the inner areas of the spheroids become hypoxic and hyponutrient as the spheroids grow. Therefore, numerous studies have been conducted to fabricate channels for perfusion through cell aggregates and cultured tissues to distribute nutrients and oxygen [2,3,4,5,6,7]. However, in cancer cell research, it is crucial to study cells under hypoxic and hyponutrient conditions. The purpose of this study was to develop a device that forms cancer cell aggregates based on cell-to-cell adhesion (similar to the formation of spheroids) and to create a hypoxic and hyponutrient region in an area that can be observed from the bottom of the chamber.

In tumors in vivo, oxygen and nutrient concentrations decrease further away from the blood vessels. Cancer cells in a hypoxic and hyponutrient region are more susceptible to metastasis and less sensitive to chemotherapy and radiation therapy [8,9]. Consequently, patient prognoses and outcomes are adversely affected. Therefore, it is desirable to reproduce hypoxic and hyponutrient regions in cancer cell cultures. However, in traditional spheroids, hypoxic and hyponutrient regions cannot be observed from the outside because they are formed inside the cell mass [10]. For example, in the case of HCT116 cells derived from human colon cancer (used in this study), a hypoxic and hyponutrient region was formed at depths of ≥100 μm from the surface. Therefore, confocal microscopy cannot be used [11]. Two-photon microscopy enables deeper observation; however, it is very expensive and not commonly used [12,13]. Although transparent reagents can be used to observe the inner parts of spheroids [14,15,16], formalin fixation is required, thus making live cells impossible to observe.

Microfluidic devices have been developed to create nutrient and oxygen concentration gradients to culture cells in two dimensions [17,18]. Two-dimensionally cultured cells exposed to nutrient and oxygen concentration gradients can be easily observed by creating a planar concentration gradient in the cell culture chamber. A microfluidic tumor slice model has been developed to form nutrient concentration gradients in microfluidic channels [19]. A polydimethylsiloxane (PDMS) rod was placed in the chamber, and a nutrient supply channel was created by removing the rod after the collagen gel-containing cells in the chamber solidified. A spatial gradient of nutrients and oxygen was generated as a function of the distance from the channel. However, owing to the low-cell density of the collagenous scaffold, the diffusion distance between the nutrients and oxygen was large, which resulted in a hypoxic and hyponutrient region at 4 mm from the supply channel. This was different from the cell aggregation achieved via cell-to-cell adhesion in spheroids. Cell density is known to affect cell morphology [20], and drug efficacy is thought to be affected by cell density. Most flow channels created in cell masses or cultured tissues use gels composed mainly of extracellular matrix components, such as collagen gels, to solidify cells for the fabrication of cell aggregates; these aggregates are then used to create cavities through sacrificial layers. Studies reported in [2,3,4,5,6,7] also used collagen gels to solidify cells and create cavities for perfusion flow channels using sacrificial layers, which implies the cell density and cell adhesion conditions were different from those in the spheroid. Therefore, these techniques were not used in the present study.

Another approach involves the use of vascular cells as perfusion channels [21,22,23,24,25,26,27,28,29]. Microfluidic channels that supply the medium are connected to the spheroid by vascular cells cultured in the area between the channels and spheroid [21,22]. However, this method requires advanced cell culture techniques. Research is being conducted to reproduce the vascular wall in a sophisticated manner [23,24,25,26,27,28,29]. However, these studies aim to clarify the nature of vascular cells inside cancer tissues. Nutrient and oxygen concentration gradients were not necessarily generated at the desired locations because the location of vascularization could not be controlled.

Thus, a device for observing nutrient and oxygen concentration gradients in cell aggregates created by cell–cell adhesion without the use of extracellular matrix gels has not yet been created. Therefore, our purpose is to observe the gradient of nutrient and oxygen concentration in cell aggregations achieved via cell-to-cell adhesion, similar to that of conventional spheroids. To this end, we proposed the use of alginate gel fibers to ensure a flow path for supplying nutrients and placed the alginate gel fibers close to the bottom part of the chamber. Herein, we report a method for their fabrication. Alginate gels are used in many cell culture devices [30,31,32,33,34,35,36] and can be used without affecting the cell cultures [36,37].

## 2. Materials and Methods

### 2.1. Device Design

The desired cell aggregates can be created from cell suspensions or by culturing spheroids in the usual manner and then introducing them into the cell culture chamber of the device. For cell suspensions, a sloped surface on which the cells can slide easily is required. Owing to the cost involved, we created the desired cell aggregates by introducing spheroids. For this, the bottom of the culture chamber can be flat.

**Figure 1:**
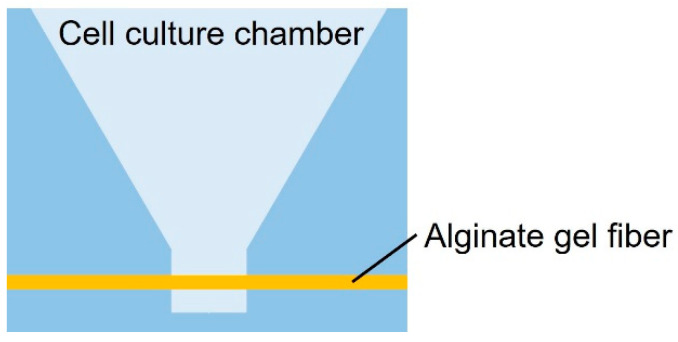
Schematic of the cell culture chamber.

**Figure 2:**
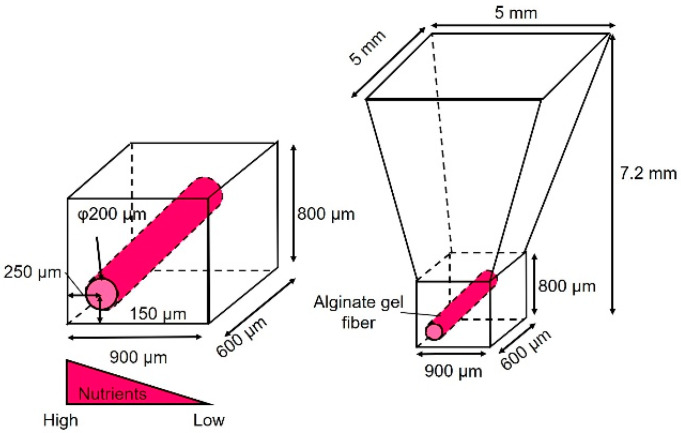
Dimensions of the cell culture chamber.

As shown in Figure 1, the cancer cell aggregate culture device consists of a cell culture chamber used to introduce spheroids to form cell aggregates and an alginate gel fiber to supply nutrients. The geometrical characteristics of the chamber are shown in Figure 2. The rectangular part represents the cell culture section where the introduced spheroid falls owing to its weight. To supply nutrients, alginate gel fibers were placed through cell aggregates to form a nutrient gradient on one side. The rectangular cell aggregates were 600 µm in width, 900 µm in depth, and 800 µm in height. The device was placed in a Petri dish filled with a culture medium to supply nutrients. A reservoir for the culture medium was prepared inside the device, thus assuming that the device could be removed from the Petri dish.

### 2.2. Device Fabrication Procedure

#### 2.2.1. Alginate Gel

The alginate gel fiber cannot be fabricated externally and then placed into the device. Therefore, we developed a method to fabricate the alginate gel fiber inside the device. Gel fabrication inside the device requires an adjustable alginate gelation rate. This is because if the gelation rate is too high, the alginate gels before it is injected into the device, which prevents its injection, and if the rate is too low, the gel injected into the device leaks before it becomes effective. In this study, we delayed the gelation by using calcium carbonate, which shows very low solubility in water [38]. Alginate is an insoluble intercellular polysaccharide derived from seaweeds. Carboxyl groups in alginate molecules react with multivalent cations, such as Ca^2+^, and gelation occurs when a specific concentration is reached. Alginate gels are typically prepared by reacting a sodium alginate solution with an aqueous Ca^2+^ chloride solution. However, this method is not suitable for gelation in devices because the gelation occurs immediately when sodium alginate and calcium chloride come into contact.

In the calcium carbonate method, a gel may form after mixing the sodium alginate solution, calcium carbonate suspension, and carbonated water. Calcium carbonate is insoluble in pH 7 water; however, as the pH decreases owing to mixing with carbonated water, Ca ions are gradually released, and gelation proceeds. By injecting a pregelled mixed solution (which remains in liquid form) into the channel, the channel can be filled with gel. By adjusting the pH through the amount of water carbonate in the alginate gel solution, the amount of Ca^2+^ released from the calcium carbonate could be adjusted, thus delaying the gelation and enabling the alginate gel to be introduced into the device. The channel connected to the chamber was not completely filled with alginate gel, thus owing to the shrinkage of the alginate gel during gelation. However, the alginate gel fiber prevents the cells from migrating, as they clog the channel when the cell aggregate is created from the cell suspension. When the cell aggregate is created from a spheroid, the alginate gel is necessary to ensure a flow path in the chamber by preventing the spheroid from filling the chamber as it grows.

#### 2.2.2. Device Fabrication

**Figure 3:**
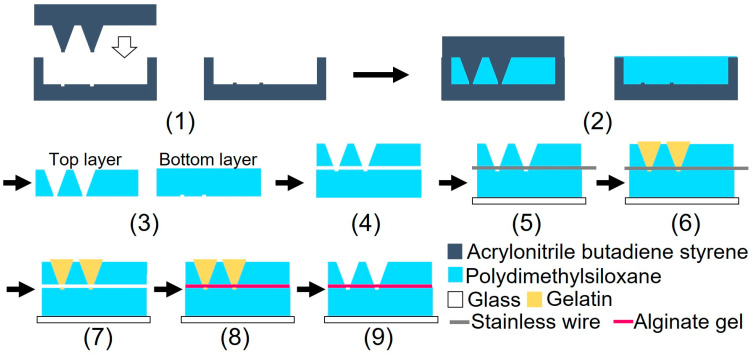
Schematic of the device fabrication processes.

Figure 3 illustrates the device fabrication process. The device was fabricated using biocompatible PDMS, which is permeable to oxygen. A glass slide was bonded to the bottom part of the device to prevent oxygen permeation. The device consisted of three parts: top, bottom, and alginate gel fiber. Although heating for 1 h was required to bond the top and bottom parts of the device, the alginate fiber was characterized by its tendency to dry easily. The dried alginate gel fiber was brittle when immersed in water. To prevent the alginate gel fiber from drying out, a sacrificial layer of gelatin was used to form the gel fiber inside the device after device assembly.

**Figure 4:**
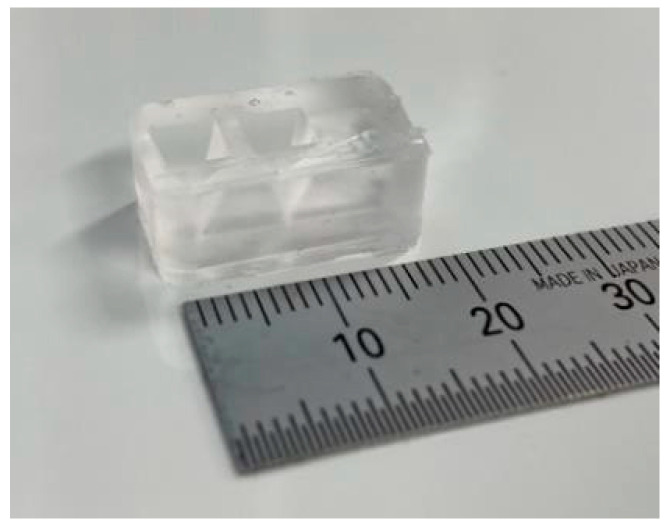
Cell culture device.

(1) Three molds were fabricated by cutting acrylonitrile butadiene styrene using a 3D modeling machine (Modela MDX-40A, Roland DG, Hamamatsu, Shizuoka, Japan). (2) PDMS was poured into the mold and cured in a furnace at 80 °C for 1 h. (3) After curing, the PDMS parts were released from the mold. (4) The bonding surfaces of the top and bottom PDMS parts were hydrophilized during 55 s using an excimer lamp (Min-Excimer, USHIO Inc., Chiyoda, Tokyo, Japan) to be bonded together. (4) The top and bottom PDMS parts were bonded by heating at 120 °C for 1 h. (5) The bottom surfaces of the device and glass slide were irradiated with an excimer lamp for 55 and 180 s, respectively, and the glass slide was bonded to the bottom surface of the device. Furthermore, a 0.2 mm stainless steel wire (Samini, Hamamatsu, Shizuoka, Japan) was inserted into the cavity. (6) A gelatin solution (Morinaga Seika, Tokyo, Japan), 20 g/L sodium alginate solution (Wako Pure Chemicals, Osaka, Osaka, Japan), and 30 g/L calcium carbonate suspension (Hayashi Pure Chemicals Industries, Osaka, Osaka, Japan) were prepared. The solutions and the PDMS device were sterilized using an autoclave (KTS-2322, Alp Inc., Minato, Tokyo, Japan). The gelatin solution was introduced into the two chambers using a syringe and cooled on ice for 4 h. (7) After the gelatin was cured, the stainless steel wire was removed. (8) An alginate gel solution was prepared by mixing 10 mL of sodium alginate solution, 7.5 mL of calcium carbonate suspension, and 8.0 mL of carbonated water (Asahi Beverage, Sumida, Tokyo, Japan). To sterilize the device, the procedures described in step (6) were performed on a clean bench. The alginate gel solution was injected into the channel using a syringe and maintained at 20 °C for 1 h. (9) The gelatin was removed by immersing the device in the DMEM (Dulbecco’s Modified Eagle’s Medium) and was incubated at 37 °C for 2 nights. Figure 4 shows a photograph of the fabricated device.

### 2.3. Experimental Setup

#### 2.3.1. Confirmation of the Effects on Cells by Changing the Alginate Gel Material

All recombinant DNA experiments were performed with the approval of the recombinant DNA advisory committees of the Tokyo Institute of Technology. All methods were performed in accordance with relevant guidelines and regulations. Human colorectal carcinoma cell line HCT116 and human glioblastoma cell line U-87MF were purchased from the American Type Culture Collection (ATCC, Manassas, VA, USA). An HCT116 subline that stably carries a fluorescent ubiquitination-based cell cycle indicator (FUCCI), HCT116/FUCCI, was established after lentiviral transduction of the gene encoding mKO2-hCdt1 (30/120) and mAG-hGeminin (1/110) in pFucci-G1 Orange and pFucci-S/G2/M Green-Hyg vectors (MBL, Nagoya, Aichi, Japan), respectively, into HCT116 cells. These cells were maintained in a 5% CO_2_ incubator at 37 °C with 5% FBS-DMEM medium (Thermo Fisher Scientific, Waltham, MA, USA). Penicillin (100 U/mL) and streptomycin (100 μg/mL) (Nacalai Tesque, Chukyo, Kyoto, Japan) were added to all media.

**Figure 5:**
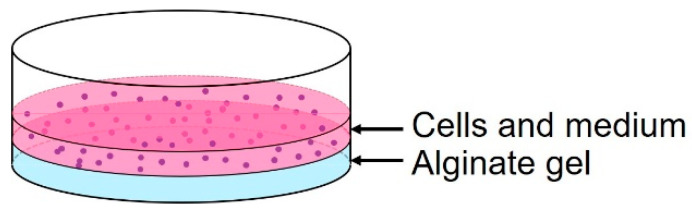
Schematic of the cell culture on alginate gel.

As shown in Figure 5, the cells were cultured on an alginate gel layer in a Petri dish to investigate the effects of alginate gel material changes. The conventional method (20 g/L sodium alginate solution + 20 g/L calcium chloride solution (Wako Pure Chemicals, Osaka, Osaka, Japan)) and the proposed method (20 g/L sodium alginate solution + 30 g/L calcium carbonate suspension + carbonated water) were used. Cancer cells (U-87MG) were cultured on each alginate gel for 5 d, and the survival rates were compared.

The viability of the cancer cells was assessed using the following procedure. First, the culture medium in a Petri dish was centrifuged to collect the cells. After washing the cancer cells with physiological saline (Wako Pure Chemicals, Osaka, Osaka, Japan), 100 µL of Calcein-AM (Wako Pure Chemicals, Osaka, Osaka, Japan) (2 µmol/L), a fluorescent dye for staining viable cells, propidium iodide (Wako Pure Chemicals, Osaka, Osaka, Japan) (3 µmol/L), and a fluorescent dye for staining dead cells were mixed with the cancer cells and kept in a CO_2_ incubator for 30 min. A small suspension aliquot volume (10 μL) was dropped onto a glass slide and covered with a cover glass. Samples were observed under an inverted fluorescence microscope (Axio Vert 100; Carl Zeiss, Oberkochen, BW, Germany). A universal serial bus microscopy camera (WRAYCAM-VEX230M, Wraymer, Osaka, Osaka, Japan) was used to acquire microscopic images, and ImageJ (version 1.54g, National Institutes of Health, Bethesda, MD, USA) was used to adjust the contrast and superimpose the images of live (green filter) and dead (red filter) cancer cells. The numbers of live and dead cancer cells were measured, and the survival rate was investigated.

#### 2.3.2. Cultivation of Cancer Cell Aggregates and Evaluation of Their Nutritional Status

HCT116/FUCCI cells were cultured on the device. HCT116/FUCCI cells fluoresce at an excitation wavelength of 561 nm during the G1 phase of the cell cycle and at an excitation wavelength of 488 nm during the S/G2 phase [39,40]. The cells in the transition stage from the G1 to S/G2 phase fluoresce at both wavelengths; therefore, they should be included as S/G2 cells. The areas with a relatively high proportion of the former are likely to be dormant areas, hypoxic and hyponutrient areas, and those of the latter are likely to be areas of growth with high oxygen and nutrients. The spheroids were formed by culturing HCT116/FUCCI cells for 2 d in U-shaped 96-well culture plates. The cell density of the cell suspension was 1 × 10^4^ cells/mL. The average diameter of the spheroids introduced into the chamber was approximately 300 μm, which was about half the width of the chamber to facilitate the introduction.

First, a spheroid and DMEM were introduced into the cell culture chamber; the device was incubated until the spheroid spread throughout the chamber. After the spheroids spread, covered the full width of the chamber, and covered or reached the alginate gel fibers, they were observed using an inverted confocal microscope.

**Figure A1:**
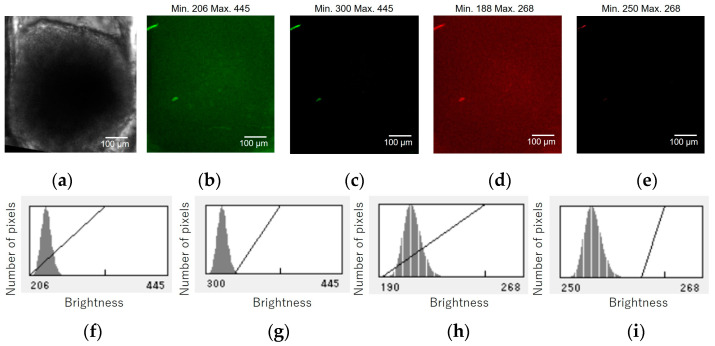
Micrographs and graphs of the HCT116/non-FUCCI spheroid in the cell culture chamber of the device. (**a**) Bright field micrographs of the HCT116/non-FUCCI spheroid in the device chamber. (**b**–**e**) Green and red fluorescent images at 60 µm from the bottom of the chamber before and after brightness adjustment. (**f**–**i**) Corresponding brightness histograms.

The following procedure was used for image processing and graph creation: (1) Z-slice images were acquired at 1 µm intervals using the confocal microscope. Laser excitation wavelengths of 488 and 561 nm were used, and the images were colored green and red, respectively. (2) To remove noise, the “zproject” function was used to superimpose the slices (five at a time), and an image was created by estimating the average brightness. (3) The “contrast adjustment” function was used to eliminate autofluorescence. Specifically, confocal fluorescence images of spheroids without FUCCI were acquired under the same conditions, and the maximum brightness value was obtained; this value was set as the minimum brightness value (see Figure A1 in Appendix A). (4) The green and red images were superimposed, and the “color threshold” function was used to separate the images of cells obtained under normoxic and normonutrient conditions from those under hypoxic and hyponutrient conditions. A hue value of ≥35 was considered a normoxic and normonutrient green image, whereas a hue value of ≤20 was considered a hypoxic and hyponutrient red image.

## 3. Results

### 3.1. Confirmation of the Effects on Cells by Changing Alginate Gel Material

**Figure 6:**
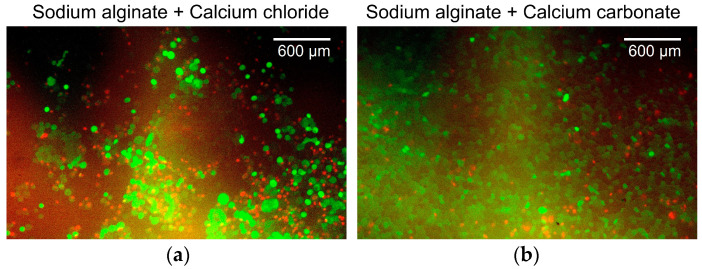
Overlaid green/red fluorescent images of the cells. (**a**) Cancer cells cultured on alginate gels prepared with aqueous sodium alginate and calcium chloride solutions. (**b**) Cancer cells cultured on alginate gels prepared from aqueous sodium alginate solution, calcium carbonate suspension, and carbonated water.

Figure 6 shows the overlaid green/red fluorescent images of the cancer cells. Figure 6a shows the cancer cells cultured on alginate gels prepared with aqueous sodium alginate and calcium chloride solutions. The survival rate of the cancer cells was 41%. Figure 6b shows cancer cells cultured on alginate gels prepared from an aqueous sodium alginate solution, calcium carbonate suspension, and carbonated water. The survival rate of the cancer cells was 72%. Both survival rates were low because the surface of the alginate gels had low cell adhesiveness, which is not the ideal environment for the culture of the adherent cells. In addition, because live cells were identified by staining the cell membrane, the boundaries between the cells could not be determined when cells were aggregated. These aggregates were counted as a single cell. This resulted in a lower estimate of the number of viable cells, whereas dead cells were counted more accurately because they were identified by staining the cell nucleus. As no substantial change was observed in the survival rate, changing the alginate gel preparation method was considered to have a minor effect on the cancer cell culture in the device.

### 3.2. Cultivation of Cancer Cell Aggregates and Evaluation of Their Nutritional Status

**Figure 7:**
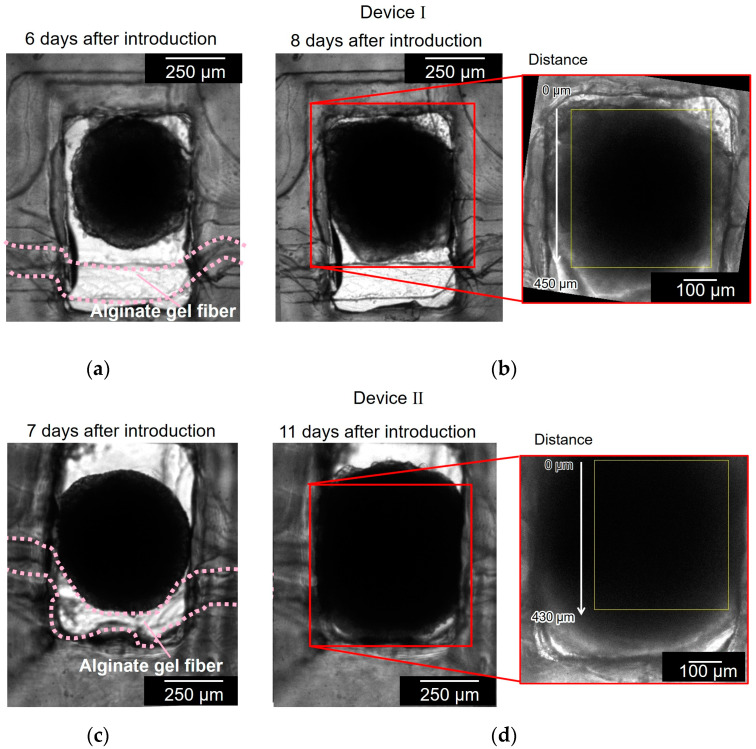
Bright field micrographs of the cell aggregates in two different devices (devices I and II). (**a**,**b**) Cell aggregate in device I before and after the cell aggregate had spread to the full width of the chamber and reached to the alginate gel fiber; (**c**,**d**) cell aggregate in device II before and after the cell aggregate had spread to the full width of the chamber and covered the alginate gel fiber.

Figure 7 shows the cell aggregates in two different devices (devices I and II). Figure 7a,b show the cell aggregate in device I before and after the cell aggregate had spread to the full width of the chamber and reached the alginate gel fiber. It took 8 d to grow the spheroid to reach the alginate gel fiber. Figure 7c,d show the cell aggregate in device II before and after the cell aggregate had spread to the full width of the chamber and covered the alginate gel fiber. It took 11 d to grow the spheroid to reach the alginate gel fiber. As observed in Figure 7a,c, the alginate gel fibers were bent in the chamber because of the unnecessary space between the fiber and chamber wall; the alginate gel fibers should have been placed closer to the chamber wall.

**Figure 8:**
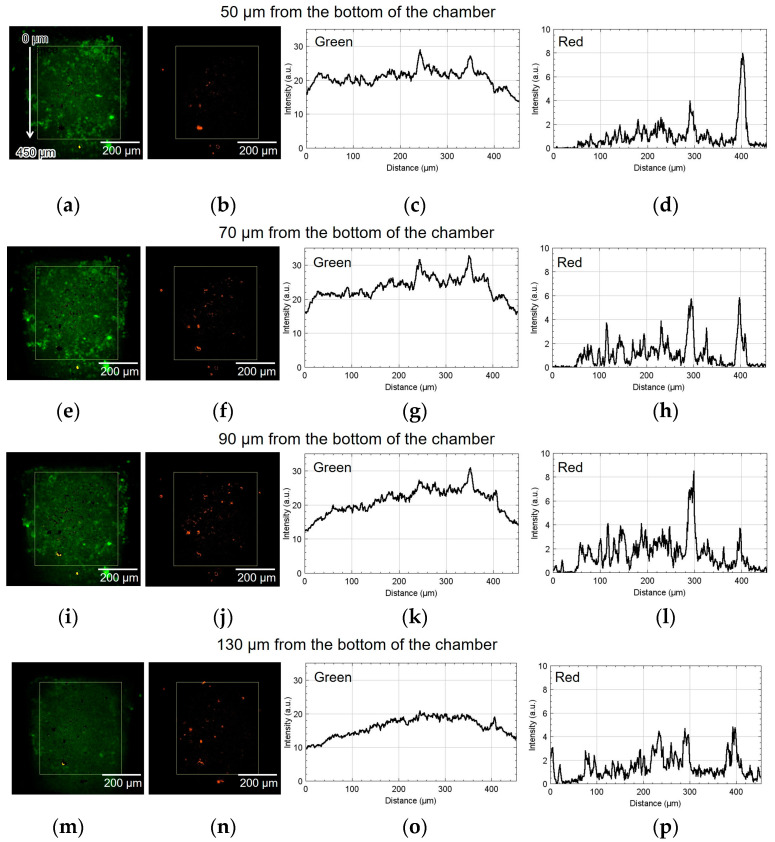
Micrographs and graphs of the cell culture chamber of device I. (**a**,**b**) Green and red fluorescent images at 50 µm; (**c**,**d**) corresponding analyzed graphs of their intensities along the vertical direction; results at (**e**–**h**) 70, (**i**–**l**) 90, and (**m**–**p**) 130 µm.

Figure 8 shows the result of using device I. The spatial distribution of the fluorescence intensity in the yellow rectangle shown in Figure 8a,b was measured. The origin of the vertical coordinate is at the top of the yellow rectangle: the larger the coordinate value, the closer it is to the alginate gel fiber. Figure 8a,b show green and red fluorescent images processed by ImageJ (NIH). Figure 8c,d depict the graphs of their intensities along the vertical direction, thus showing the difference in luminescence corresponding to the position of the alginate gel fiber. The approximate height from the bottom of the chamber was 50 µm. The cell aggregate was also observed at 70, 90, and 130 µm from the bottom of the chamber. Similarly, Figure 8e–h, Figure 8i–l, and Figure 8m–p show the results at 70, 90, and 130 µm, respectively.

As shown in Figure 8h, the number of cells emitting red fluorescence indicating hypoxia and hyponutrition increased in the area far from the alginate gel fiber compared to that shown in Figure 8d. As shown in Figure 8k, the number of cells emitting green fluorescence indicating normoxia and normonutrition decreased in the area far from the alginate gel fiber compared to that shown in Figure 8g. Therefore, at heights of 70 and 90 μm, a vertical nutritional gradient between normoxic and normonutrient regions and hypoxic and hyponutrient regions might be observed when spheroid-based cancer cell aggregates are cultured using this device. Figure 8o,p show that both the green and red signals decayed at the height of 130 μm.

**Figure 9:**
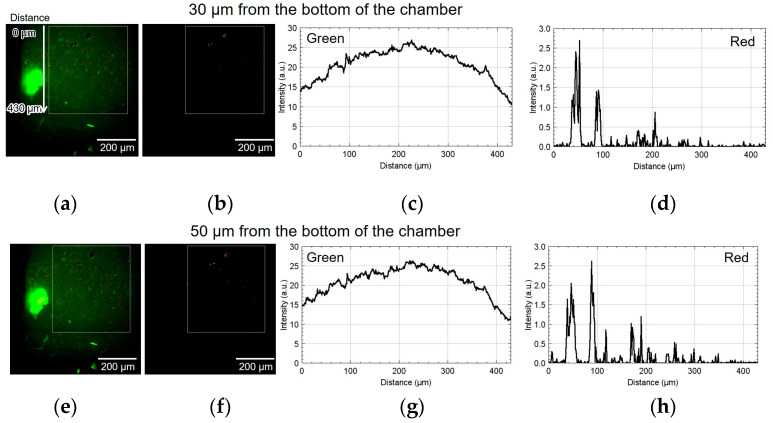

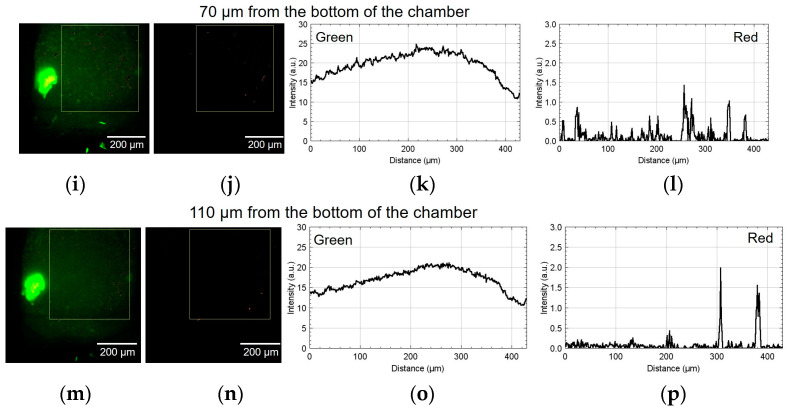
Micrographs and graphs of the cell culture chamber of device II. (**a**,**b**) Green and red fluorescent images at 30 µm; (**c**,**d**) corresponding analyzed graphs of their intensities; results at (**e**–**h**) 50, (**i**–**l**) 70, and (**m**–**p**) 110 µm.

Figure 9 shows the result of using device II. Figure 9a,b show the green and red fluorescent images at 30 µm from the bottom of the chamber. Figure 9c,d show the analyzed graphs of their intensities. Similarly, Figure 9e–h, Figure 9i–l, and Figure 9m–p show the results at 50, 70, and 110 µm, respectively. At the heights of 30 and 50 μm, the number of cells emitting red fluorescence indicating hypoxia and hyponutrition increased in the area far from the alginate gel fiber, and the number of cells emitting green fluorescence indicating normoxia and normonutrition decreased in the area far from the alginate gel fiber; gradients of oxygen and nutrient concentrations were generated at a height of 30–50 μm. At a height of 70 μm, the green fluorescence was slightly reduced; however, the red fluorescence increased throughout the region. Furthermore, at a height exceeding 70 μm, the green fluorescence decreased, thus suggesting that the entire region became hypoxic and hyponutrient because it was far from the alginate gel fiber. Figure 9o,p show that the green signals decayed, and the red signals far from the gel fiber decayed at the height of 110 μm.

In device I, the position at 450 μm was approximately 50 μm away from the center of the alginate gel fiber. In device II, the position at 430 μm was approximately 50 μm away from the center of the alginate gel fiber. Thus, in both cases, the hypoxic and hyponutrient region were located approximately 500 µm from the alginate fiber.

## 4. Discussion

We fabricated a cancer cell aggregate culture device that facilitated the observation of nutrient and oxygen concentration gradients using an alginate gel fiber. Spheroids were introduced into and cultured in a chamber where an alginate gel fiber was placed. The experimental results suggested the presence of concentration gradients. A hypoxic and hyponutrient region was created at a distance of approximately 500 µm from the alginate gel fiber. Conventional 3D cell culture devices use extracellular matrices, such as collagen. In [19], the hypoxic and hyponutrient region was created at distances of 4 mm from the culture medium supply channel. Our device has a sufficiently short diffusion distance owing to the cell aggregation achieved via cell-to-cell adhesion in the spheroids.

The current limitation of this study was the inability to control and position the spheroid relative to the alginate gel fiber when introducing it into the chamber. To overcome this limitation, designing a device in which cells aggregate at a predetermined position relative to the alginate gel fiber by culturing the cell aggregate from cell suspension will be an effective solution.

## 5. Conclusions

In this study, an alginate gel fiber was used to ensure a flow path for supplying nutrients and oxygen to the spheroids. This method could be applied to supply not only nutrients and oxygen but also drugs. In the present study, only one fiber was placed; however, nutrient and oxygen concentration gradients can be created in larger 3D cultured cells by placing multiple fibers. Thus, the method of arranging alginate gel fibers in a channel is more scalable than the conventional method of supplying nutrients and oxygen using diffusion in a spheroid culture.

## Data Availability

The data that support the findings of this study are available from the corresponding author upon reasonable request.

## Appendix A

Confocal fluorescence images of a HCT116/non-FUCCI spheroid were acquired under the same conditions. Figure A1 shows the micrographs and histograms of the HCT116/non-FUCCI spheroid in the cell culture chamber of the device. Figure A1a shows bright field micrographs of the HCT116/non-FUCCI spheroid in the device chamber. Figure A1b–e show the green and red fluorescent images at 60 µm from the bottom of the chamber before and after brightness adjustment. The corresponding histograms are shown in Figure A1f–i. The brightness adjustments suggest that the brightnesses of the green and red autofluorescence of the HCT116/non-FUCCI spheroid were 300 and 250, respectively.
